# Now for the Queen City of the Lakes

**Published:** 1896-07

**Authors:** 


### NOW FOR THE QUEEN CITY OF THE LAKES.

On other pages will be found a synopsis of the programme for our Buffalo meeting in September next, and it is sufficiently well advanced to offer something to attract every veterinarian throughout the country. Its variety of subjects, not to mention the one great field of sanitary work and its branches of milk- and meat-inspection, toward which every eye is turned and which commands the attention of every veterinarian, because he knows not the day or the hour he may be called to fill a post of this character in his town or city, must bring to this convention an unusual attendance. These subjects are so new in a sense that the text-books of ten years ago are of little value now, and this progress is added to day by day, so that one is compelled to mingle with the most progressive spirits of this great movement to be able to properly advise with and counsel his people in measures destined to wisely control and care for these interests. The work of Secretary Stewart is well in hand, and every means to make this the best meeting in our Association's history are being put in force by him and must command the hearty co-operation of every member.

A welcome to all at Buffalo.

